# Transcriptomic profiling of a chicken lung epithelial cell line (CLEC213) reveals a mitochondrial respiratory chain activity boost during influenza virus infection

**DOI:** 10.1371/journal.pone.0176355

**Published:** 2017-04-25

**Authors:** Léa Meyer, Olivier Leymarie, Christophe Chevalier, Evelyne Esnault, Marco Moroldo, Bruno Da Costa, Sonia Georgeault, Philippe Roingeard, Bernard Delmas, Pascale Quéré, Ronan Le Goffic

**Affiliations:** 1 VIM, INRA, Université Paris-Saclay, Jouy-en-Josas, France; 2 ISP, INRA, Université François Rabelais de Tours, UMR 1282, Nouzilly, France; 3 Centre de Ressources Biologiques pour la Génomique des Animaux Domestiques et d’Intérêt Economique, CRB GADIE INRA, Domaine de Vilvert, Jouy-en-Josas, France; 4 Plateforme IBiSA de Microscopie Electronique, Université François Rabelais and CHRU de Tours, Tours, France; 5 INSERM U966, Université François Rabelais and CHRU de Tours, Tours, France; University of California Davis, UNITED STATES

## Abstract

Avian Influenza virus (AIV) is a major concern for the global poultry industry. Since 2012, several countries have reported AIV outbreaks among domestic poultry. These outbreaks had tremendous impact on poultry production and socio-economic repercussion on farmers. In addition, the constant emergence of highly pathogenic AIV also poses a significant risk to human health. In this study, we used a chicken lung epithelial cell line (CLEC213) to gain a better understanding of the molecular consequences of low pathogenic AIV infection in their natural host. Using a transcriptome profiling approach based on microarrays, we identified a cluster of mitochondrial genes highly induced during the infection. Interestingly, most of the regulated genes are encoded by the mitochondrial genome and are involved in the oxidative phosphorylation metabolic pathway. The biological consequences of this transcriptomic induction result in a 2.5- to 4-fold increase of the ATP concentration within the infected cells. PB1-F2, a viral protein that targets the mitochondria was not found associated to the boost of activity of the respiratory chain. We next explored the possibility that ATP may act as a host-derived danger signal (through production of extracellular ATP) or as a boost to increase AIV replication. We observed that, despite the activation of the P2X7 purinergic receptor pathway, a 1mM ATP addition in the cell culture medium had no effect on the virus replication in our epithelial cell model. Finally, we found that oligomycin, a drug that inhibits the oxidative phosphorylation process, drastically reduced the AIV replication in CLEC213 cells, without apparent cellular toxicity. Collectively, our results suggest that AIV is able to boost the metabolic capacities of its avian host in order to provide the important energy needs required to produce progeny virus.

## Introduction

Avian influenza viruses (AIVs) are enveloped, single-stranded, negative-sense RNA viruses which belong to the *Orthomyxoviridae* family. They can cause disease in poultry and wild birds, but also in humans. AIVs represent a major source of epizootic outbreaks that seriously impacts local and international trade [1]. In addition to economic costs, highly pathogenic (HP) AIVs are also a serious threat for human health due to the capacity of certain strains to cross species barriers and cause human infections [2]. Within its avian host, HPAIVs can replicate in a wide variety of tissues and cell types and induce severe systemic disease in chickens with very high mortality. By contrast, low pathogenic (LP) AIVs mainly replicate in epithelial tissue, principally respiratory and digestive [3]. LPAIVs cause asymptomatic to mild diseases but induce a decrease in growth performance and in egg production [3].

Among birds, the fecal-to-oral transmission is the most common type of spread. However, in modern poultry farms, thousands of birds are concentrated in grow out houses, facilitating the aerosol transmission of the AIVs through the flock. The epithelial airways of the chickens are thus at the front line in terms of defense against AIVs. The innate immune response of epithelial cells is therefore of first importance to limit the spreading of the virus throughout the animal. Early immune responses have been described in tracheal epithelial cells infected by AIV [4]. The defense response is rapidly induced, involving the expression of chemokines, antiviral cytokines and gallinacin genes, demonstrating that the tracheal epithelial and mucosal cells are able to mount an innate immune response against the AIVs [5]. Although the tracheal cells are anatomically the nearest AIVs targets during aerosol contamination of chickens, the unidirectional air flow produced during the birds respiratory cycle also allows the infection of lung capillaries [6], making the lung epithelium another primary target of the AIV.

In chickens, the recognition of AIVs infection is not mediated through the RIG-I (retinoic acid-inducible gene I) helicase since the gene encoding this protein is absent [7]. This specificity is a plausible explanation for the high susceptibility of chickens to AIVs. As a matter of comparison, ducks express RIG-I and are described as highly resistant to AIVs infections, although the late 2016 HPAIV H5N8 outbreak seems particularly virulent in ducks. Still, the chicken cytosolic helicase Melanoma Differentiation-Associated protein 5 (MDA5) is able to sense pathogen associated molecular patterns (PAMPs) associated to AIVs, and thus to compensate for the absence of RIG-I. Importantly, this pattern recognition receptor is targeted by the AIV viral protein non-structural 1 (NS1) [8]. Once activated by its viral RNA ligand, MDA5 mediates a signaling through the Mitochondrial antiviral-signaling protein (MAVS) which activates the Interferon regulatory factor 7 (IRF7) and nuclear factor-kappa B (NF-kB) pathways. Indeed, mitochondrion plays the role of a docking station for the multiprotein complex leading to their activation. Beyond its role of adaptor of the antiviral signaling, the mitochondrion is the organelle orchestrating the defense of the infected cell thanks to its critical role in apoptosis and its capacity to produce reactive oxygen species (ROS) [9]. Alongside these immune pathways, cells can react to a sudden increase of extracellular ATP, which is considered a danger signal, through the activation of the purinergic receptor P2X7 [10]. Concomitant with PAMP-mediated NF-κB activation, the activation of P2X7 receptor leads to a potent cation efflux which drives NLRP3 inflammasome assembly and thus Interleukin 1-beta (IL1-β) and Interleukin 18 (IL-18) maturation [11]. Inflammasome signaling leads to inflammation, cell death or pyroptosis [12].

In this work, we analyzed the transcriptome of a recently described cell line called CLEC213 (Chicken Lung Epithelial Cells 213) [13] infected with a low pathogenic H6N2 influenza virus. In addition to intracellular signaling and inflammatory pathway classically induced in AIVs infections, we identified an important change in mitochondrial genes expression profiles. These genes encode proteins of the oxidative phosphorylation pathway, a process that is crucial for the cell metabolism. Our data suggest that during infection of its chicken host cells, AIV is able to induce a boost in the bio-energetic capacities of the host that leads to an increase in ATP production.

## Materials and methods

### Cell lines

Chicken Lung Epithelial Cells (CLEC213) were isolated from lungs of White Leghorns chickens as described in reference [13]. The cells were maintained in Dulbecco’s Modified Eagles’s Medium (DMEM) medium supplemented with 10% Fetal Calf Serum (FCS), 2mM L-Glutamine and 100UI/ml penicillin and 100μg/ml streptomycin. Cell culture was maintained at 41°C in a 5% CO_2_ incubator.

### Viruses

Avian influenza A/turkey/Massachusetts/3740/1965 [H6N2] and A/Turkey/Italy/977/1999 [H7N1] were used in this study. H6N2 were propagated and titrated on Madin-Darby Canine Kidney (MDCK) cells. For infection, cells were washed with FCS-free Minimum Essential Medium (MEM) and incubated with virus at the indicated multiplicities of infection (MOI) diluted in FCS-free medium. The H7N1 virus was obtained using the reverse genetic system as previously described [14]. Quick-change mutagenesis kit (Stratagene, San Diego, CA, USA) was used to generate a recombinant virus containing a truncated form of Polymerase Basic protein 1-Frame 2 (PB1-F2), referred to as ΔF2 H7N1 of the A/Turkey/Italy/977/1999 [H7N1]. Briefly, a silent mutation in PB1 resulted in the introduction of a stop codon at the twelfth amino acid of PB1-F2 in order to abort its translation without affecting the other proteins of segment 2 [15].

### Nucleic acid extraction, RT and qPCR

Total RNA isolated from CLEC213 cells using the Qiagen RNeasy kit (Qiagen, Hidden, Germany) was treated with DNase I and reverse-transcribed with superscript II reverse transcriptase (Invitrogen, Carlsbad, CA, USA) using random hexamers (Thermo Fisher, Waltham, MA, USA) [16]. Total DNA was extracted from CLEC213 cells using a proteinase K digestion mix. Briefly, cells were lysed in a buffer containing Tris 100mM (pH 7,4), EDTA 5mM, SDS 0,2%, NaCl 200mM and proteinase K at 75μμg/ml. Lysates were incubated overnight at 55°C then pelleted at 13,000 rpm for 5 minutes. Supernatants were collected and DNA was precipitated with isopropanol, pelleted and washed with 70% ethanol. DNA pellets were dried and re-suspended in MilliQ water. qPCR reactions were performed in a total volume of 20 μμl with specific primer pairs for the amplification of the target chicken genes (S1 Table) and of AIV M1 [16]. The expression levels of target genes and M1 were measured using the CFX Connect qPCR platform (BioRad, Hercules, CA, USA) and the double-strand—specific dye SYBR Green kit (BioRad). The qPCR thermal cycle was as follows: initial denaturation 3 min at 95°C, followed by 40 cycles at 95°C for 10 s, followed by an annealing step at 60°C for 15 s, and then extension at 72°C during 30 s. Each point was performed in triplicate. To make sure that the primers produced a single and specific PCR amplification product, a dissociation curve was carried out at the end of the PCR cycle. Chicken genes were quantified using the comparative ΔΔCt method. The mean ΔCt obtained in mock-infected cells for each gene was used as calibrator, after normalization to endogenous control β-actin. The results are presented as an n-fold difference relative to calibrator (relative quantification = 2^-ΔΔCt^). The absolute copy number of M1 viral gene was determined using a standard curve of M1 purified plasmid. The comparison between qPCR assays and viral titrations was performed and strong correlations were obtained between the 2 viral quantifications methods (S1 Fig).

### Microarray experiments

Transcriptional profiling was performed using the Agilent chicken Gene Expression 4X44 Microarrays (AMADID: 026441, Agilent Technologies, Santa Clara, CA, USA). This microarray design is referenced within the Gene Expression Omnibus (GEO) database under the platform ID GPL15357. A dual color design was used to provide direct comparisons between infected and mock-infected CLEC213 cells. To reduce potential experimental biases, RNA samples were collected from 4 independent experiments. Arrays were hybridized according to the manufacturer’s instructions and as previously described [16]. Functional analysis of the data was done as described in reference [17]. Differentially expressed genes were identified using a moderated T-test. A Benjamini-Hochberg False Discovery Rate (FDR) was then used as multiple testing correction method and a corrected p-value cutoff of 1% was applied. A fold change >2 cutoff was then added to select the differentially expressed genes between the mock and infected conditions.

### Electron microscopy

Cells were fixed in 4% paraformaldehyde and 1% glutaraldehyde in 0.1M phosphate buffer (pH 7.3) for 48h, washed with phosphate buffer 0,15M, post-fixed in 1% osmium tetroxide for 1h and dehydrated in a graded series of ethanol solutions. Cell pellets were embedded in EPON^™^ resin (Sigma-Aldrich, Saint-Louis, MO, USA) that was allowed to polymerize at 60°C for 48h. Ultrathin sections were cut, stained with 5% uranyl acetate and 5% lead citrate and deposited on gold EM grids for examination using a JEOL 1011 transmission electron microscope.

### Cellular ATP measurement

CLEC213 cells were seeded at 1.5x10^4^ on 96-well plates in complete DMEM medium 24 h before infection. Cells were then infected with H6N2 or H7N1 (MOI = 1) in serum-free medium and lysed at different times post infection (pi) using 100μμl of CellTiter-Glo reagent (Promega, Madison, WI, USA). Live cells were counted at each time pi to determine the relative quantity of ATP per cell and thus overcome the bias introduced by the cytopathic effect (CPE) of the virus. Luciferase activity was measured in the cell lysates using a Tecan infinite M200PRO plate reader (Männedorf, Switzerland). Results are expressed as a ratio of relative luciferase units (RLU) to number of live cells.

### NF-κB activity measurement

CLEC213 cells were seeded at 6.25x10^4^ on 48-well plates in complete DMEM medium 24h before treatment. Cells were then transfected in serum-free medium using the Fugene reagent (Promega). The transfection mix contained 150ng of an NF-κB -Luciferase plasmid and 50ng of a pCI-GFP plasmid to normalize the data. Transfection mix was supplemented with ATP to hold a final concentration of 1mM or was left untreated.

### Influenza minigenome assay

CLEC213 cells were seeded at 6.25x10^4^ on 48-well plates in complete DMEM medium 24h before infection. After 24h, medium was replaced by serum-free medium and cells were left untreated or treated with 125nM oligomycin (Selleck chemicals, Houston, TX, USA) and infected or not by H6N2 (MOI = 1) in 48-well plates. Two hours pi, the cells were transfected with a plasmid expressing negative-sense firefly luciferase flanked by segment 6 noncoding sequences under the control of a chicken-specific polymerase I promoter (pgHH21-vNA-Luc, 200ng, kind gift of Dr Brown, University of Ottawa) together with a plasmid expressing beta galactosidase to normalize DNA uptake (200 ng) using Fugene HD transfection reagent (Promega). Twenty hours pi, cells were lysed with 100 μl of passive lysis buffer (25mM Tris pH 7.8, 8mM MgCl2, 1% Triton X-100, 15% glycerol, and 1mM dithiothreitol) per well for 5 min at room temperature with gentle shaking. Luciferase activity was measured in the cell lysates using a Tecan infinite M200PRO plate reader with automatic injection of 100 μμl of lysis buffer supplemented with 2mM D-luciferin (Sigma). Results are expressed as RLU relative to control.

### Statistical analyses

qRT-PCR measurements were expressed as the mean ± standard error of the mean (SEM) of at least 3 independent experiments, and statistical analyses were performed using the Student’s t-test for pair wise comparisons and ANOVA for multiple comparisons. Ontological analysis was carried out with IPA (Qiagen) and used the right-tailed Fisher’s exact test to calculate a p-value determining the probability that each biological function and disease assigned to that data set is due to chance alone. The activation status of the pathways was predicted using IPA “Upstream Regulator Analysis” tool by calculating a regulation z-score. The z-score is used to infer likely activation states of upstream regulators based on comparison with a model that assigns random regulation directions. It was considered significantly activated (or inhibited) with a z-score≥2.0 or ≤−2.0. The detailed descriptions of IPA analysis are available on the IPA website (http://www.ingenuity.com).

## Results

### Transcriptomic profiling of the CLEC213 infected by AIV

**Fig 1:**
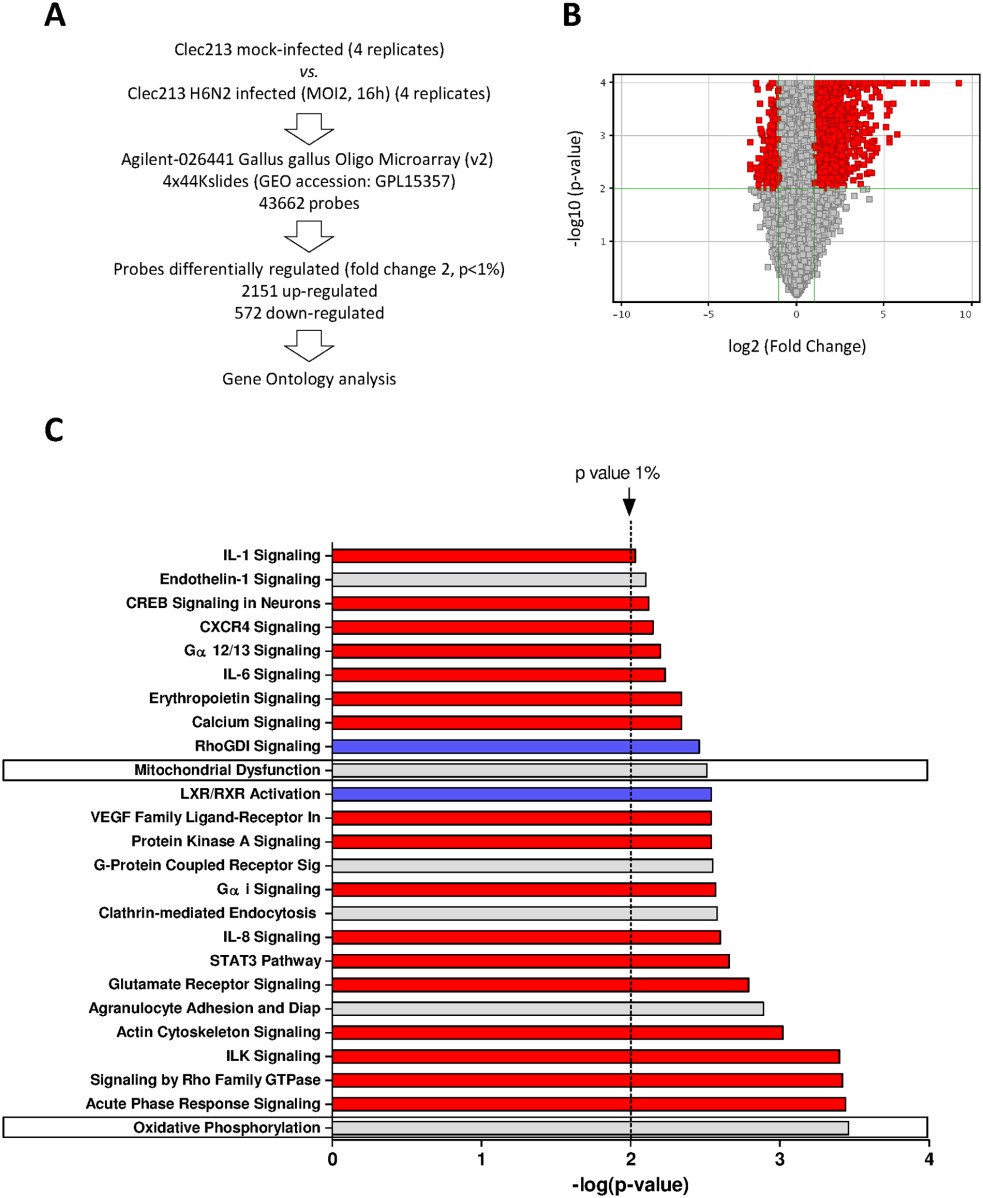
Microarray analysis of gene expression in CLEC213 cells infected with H6N2 virus. (A) Schematic representation of the experimental design and classification of differentially expressed genes. (B) Volcano plot representing the distribution of the expressed genes during the infection as a fold change (x axis) and as significance (y axis). (C) Canonical pathways associated to DEG in H6N2-infected CLEC213. Pathways were identified using IPA software and ranked with the p-value (negative logarithm of significance) obtained using the right-tailed Fisher’s exact test. Red and blue bars indicate predicted pathway activation or inhibition, respectively (z-score). Gray bars indicate pathways where no prediction can currently be made.

In order to finely characterize the response of CLEC213 cells to the influenza virus infection, we compared the transcriptomic profiles of infected and mock-infected cells using microarrays. We used the low-virulent A/turkey/Massachusetts/3740/1965 [H6N2] virus (herein named H6N2). This virus is described as inducing a moderate cytopathic effect (CPE) in cell culture [18]. CLEC213 cells were infected at a MOI of 2 for 16 hours. This time-point was chosen because the cells showed satisfactory innate immune response (preliminary qPCR data, not shown) while displaying only moderate CPE, which prevented an overrepresentation of RNA from dying cells that would have introduced a bias in the microarray. Total RNAs were then collected and processed to be hybridized to the Agilent chicken microarray. Eventually, the resulting data were processed using the GeneSpring software (Agilent). A total of 2,723 differentially expressed genes (DEGs) were obtained and used for further ontological analyses. Among these genes, 2,151 were upregulated and 572 downregulated during the viral infection (Fig 1A and 1B). We used IPA software to identify the functions and pathways associated to this set of DEG. We focused on the 25 most significantly regulated canonical pathways (Fig 1C, p-value<1%). As expected, several inflammatory pathways appeared to be induced during the AIV infection: Interleukin 1, 6, 8 (IL1, IL6, IL8) and acute phase response signaling. The cellular functions mediated through G-proteins were also predominant in the CLEC213 innate response: Gα12/13, RhoGDI, G-Protein Coupled Receptor, Gαi and Rho Family GTPases signaling. Interestingly, we also identified two functions related to the mitochondria: Mitochondrial Dysfunction and Oxidative Phosphorylation (Fig 1C, boxed pathways). Importantly, no interferon-associated pathways were identified in the frame of this experiment, suggesting that CLEC213 cells may possess an alternative antiviral response against the H6N2 virus.

### Functional relationships of virally-impacted pathways

**Fig 2:**
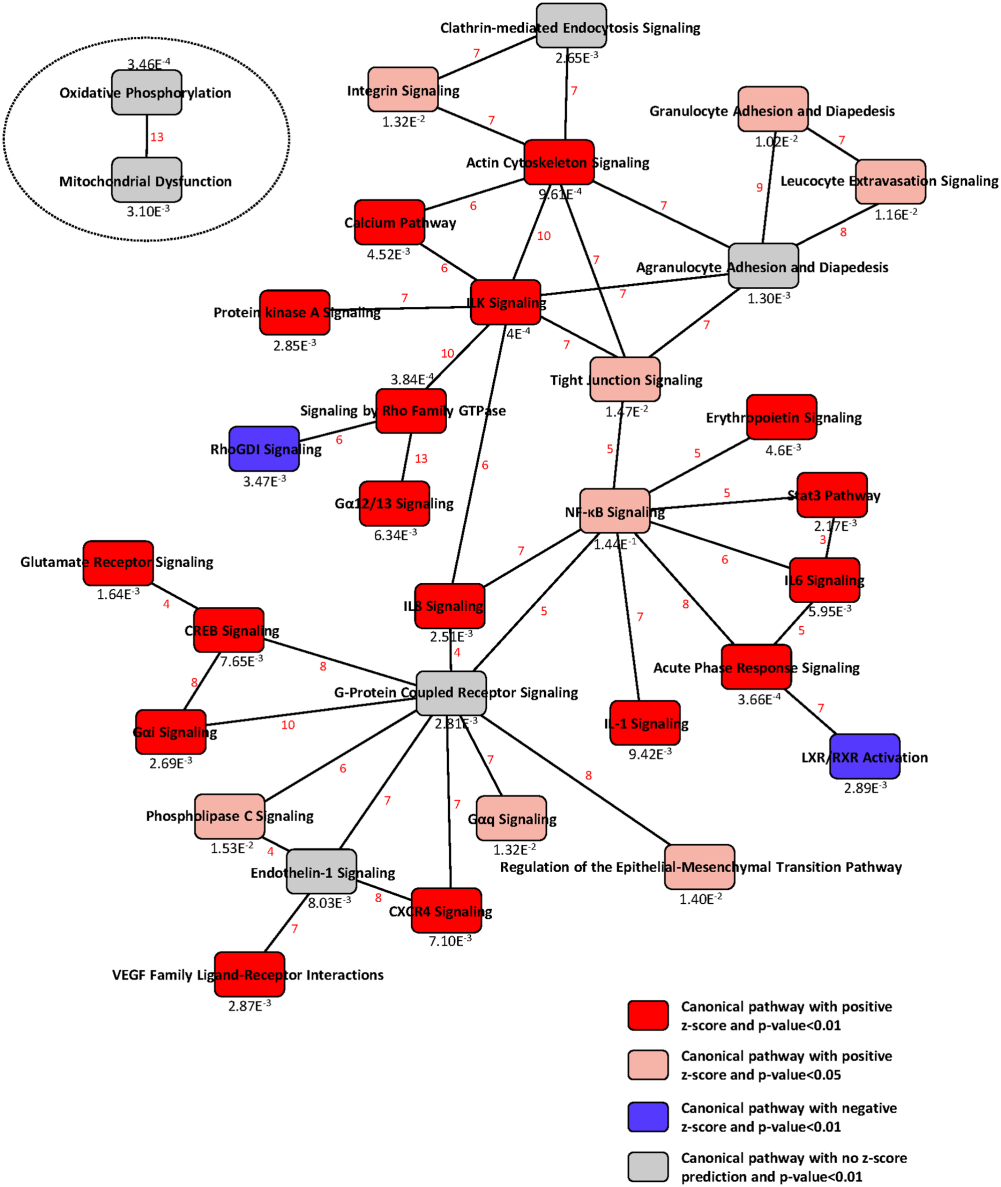
Functional relationships of pathways regulated during infection of CLEC213 with H6N2 virus. IPA software was used to build a biological network using the DEG in the H6N2-infected cells compared with mock-infected controls (twofold change, p-value<0.01). Significance of the pathways are indicated at the bottom of each node. The diagram shows the interactions between pathways. Pathways are connected through common genes; the number of shared genes between pathways is indicated in red.

In order to get a comprehensive view of the cell response, we built a network of canonical pathways, each one being linked by common DEGs. As shown in Fig 2, the majority of the virally-impacted canonical pathways formed a large integrated network. The functional analysis of this large network could be resumed in three main biological functions: inflammation, leucocytes chemoattraction and tissue morphology. Remarkably, the two mitochondrial pathways remained isolated from this large network, meaning that they shared no genes with the rest of the virally-regulated canonical pathways. This either suggests that the induction of mitochondrial genes is independent of the innate immune response of the infected cells or that it involves a mechanism yet to be characterized.

### H6N2 virus impacts the oxidative phosphorylation pathway

**Fig 3:**
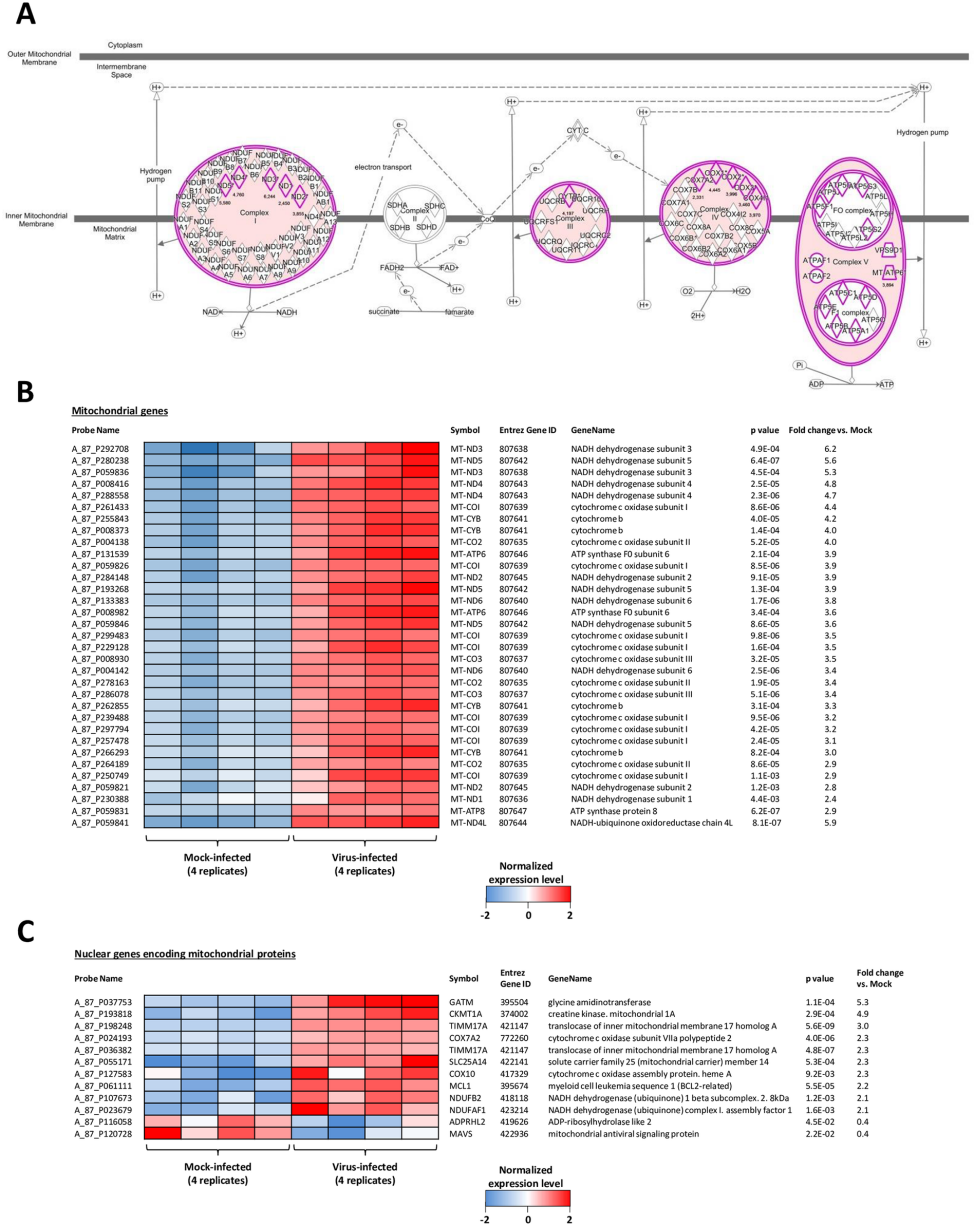
H6N2 infection impacts oxidative phosphorylation. (A) Schematic representation of the “oxidative phosphorylation” metabolic pathway, constituted of 5 multiprotein complexes. The 4 complexes induced by AIV infection are colored in pink. The detail of the proteins composing the complexes is indicated, each induced gene is highlighted in pink and the fold induction is indicated. (B-C) Heat map showing the normalized level of expression of mitochondrial DEG encoded by the mitochondrial genome (B) or encoded by the cell genome (C). The four replicates of each condition (mock- and H6N2-infected) are represented.

To understand how the H6N2 virus was able to impact mitochondria, we analyzed the Mitochondrial Dysfunction and the Oxidative Phosphorylation canonical pathways, which both mainly included DEGs implicated in the oxidative phosphorylation. These DEGs are highlighted in pink in the canonical pathway represented in Fig 3A. Oxidative phosphorylation produces ATP using the energy of an electrochemical gradient generated by the mitochondrial electron transport chain (ETC) constituted of five multiprotein complexes located within the inner mitochondrial membrane, of which 4 were impacted by the H6N2 infection (shown in pink, Fig 3A). The probes corresponding to the DEGs encoded by the mitochondrial genome are shown on the heat map of Fig 3B. In this picture, the expression levels of the four replicate samples from mock- and infected-cells are provided. All of these DEGs are covered by several probes with statistically significant p-values providing a high degree of confidence in the data. Since most of the mitochondrial proteins are encoded by nuclear genes, we also explored the regulation of nuclear-encoded mitochondrial DEGs. We identified 491 probes corresponding to nuclear genes which encode mitochondrial proteins in the chicken microarray. With the exception of 12 genes, the vast majority of these genes were not regulated more than twofold during the infection (Fig 3C). Taken together, these data suggest that the infection of CLEC213 cells with H6N2 impacts the oxidative phosphorylation metabolic pathway by inducing the expression of genes encoded by the mitochondrial genome.

**Fig 4:**
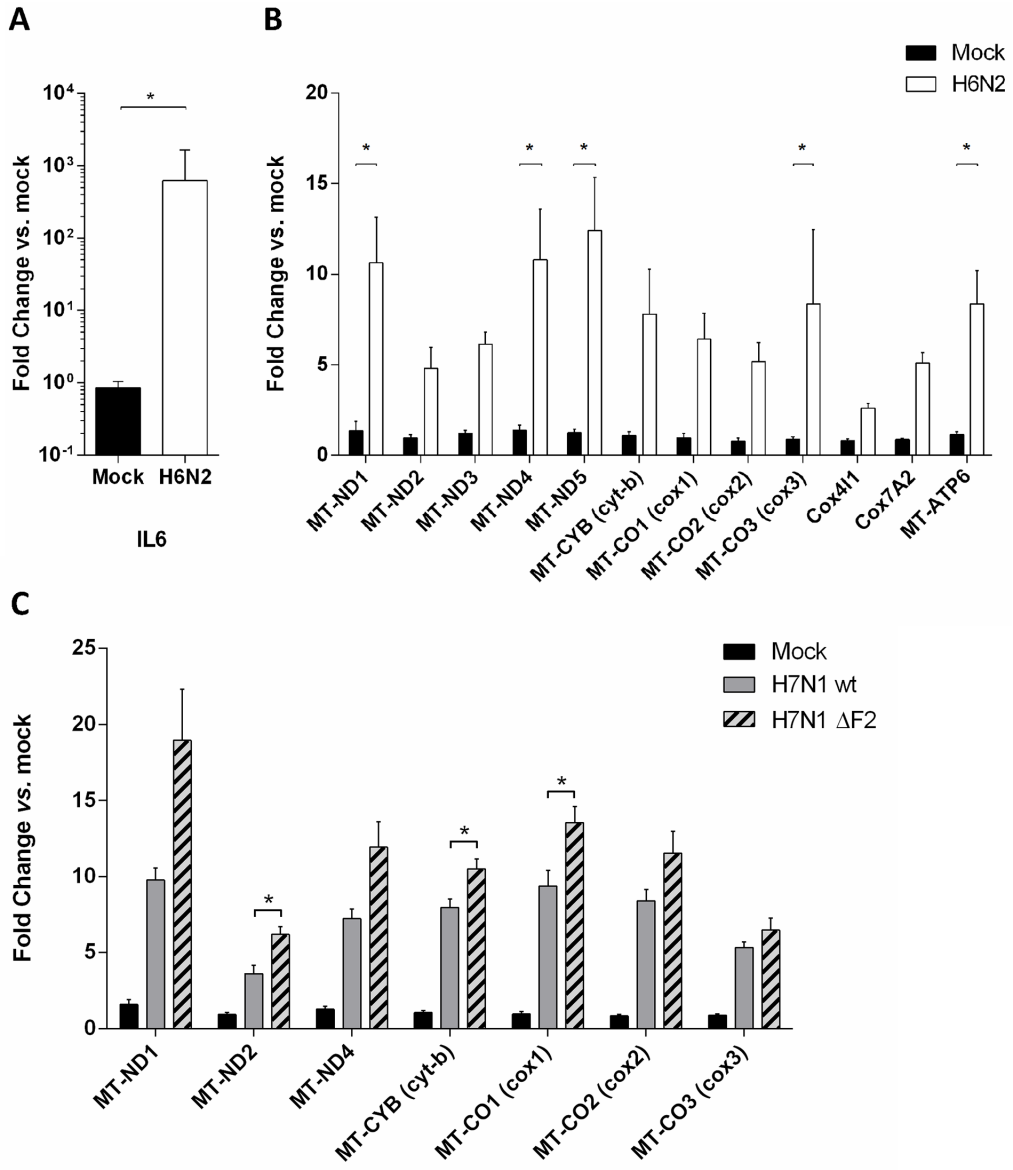
H6N2 induces an inflammatory response and a mitochondrial response at the transcriptomic level. (A) H6N2-induced inflammatory signature observed by microarray was controlled using independent samples through monitoring the level of expression of IL6 by qRT-PCR. (B) Mitochondrial genes (MT-) and mitochondria-addressed nuclear genes expression was assessed in H6N2-infected cells by qRT-PCR. (C) Mitochondrial gene expression was monitored in H7N1- and ΔF2 H7N1 infected cells. Results are expressed as means ± SEM (* p-value < 0.01).

In order to validate changes in gene expression and to confirm the microarray data, we performed qRT-PCR on independently-produced samples. The qRT-PCR assay confirmed the potent induction of inflammatory mediators with a 600 fold increase for IL6 (Fig 4A). As displayed by microarray data, the components of the oxidative phosphorylation metabolic pathway were largely induced by the H6N2 infection (Fig 4B), with slightly higher levels of expression in the qRT-PCR assay. For instance, Mitochondrially Encoded ATP Synthase 6 (MT-ATP6) was induced 8.3 fold vs. mock in qRT-PCR data and 3.9 fold in microarray data. The homogeneous regulation of the genes encoded by the mitochondria is likely due to the polycistronic organization of the mitochondrial genome [19].

### The oxidative phosphorylation metabolic pathway is targeted by other AIVs independently of PB1-F2

To further investigate the AIV-mediated increase of mitochondrial genes expression, we used another LPAIV to infect CLEC213 cells: A/Turkey/Italy/977/1999 [H7N1]. This experiment was important to exclude the possibility that the observed gene profile was strain-specific for the H6N2 AIV. In addition, we used the reverse genetics system for the H7N1 strain (a gift of Dr M. Ducatez, Toulouse) to produce a defective mutant virus, thereafter named ΔF2 H7N1, that was unable to express the viral protein PB1-F2, which is known to have a potent mitochondrial tropism [20]. CLEC213 cells were infected with WT or ΔF2 H7N1 virus (MOI = 1) and total RNA were collected at 16h pi to quantify the expression levels of mitochondrial genes. Fig 4C shows the expression profiles of the mitochondrial genes implicated in the oxidative phosphorylation metabolic pathway. Infection of CLEC213 cells with the WT H7N1 virus led to expression profiles similar to those observed for H6N2, allowing us to conclude that the observed effect is strain-independent. The ΔF2 H7N1 infection showed little to no difference with the WT virus-induced mitochondrial gene expression, indicating that PB1-F2 is not implicated in this process. The modest differences obtained between the two viruses are most likely due to the deleterious effect exerted by PB1-F2 within the mitochondrion [17, 21].

### AIV infection of CLEC213 cells induces an increase in ATP production

**Fig 5:**
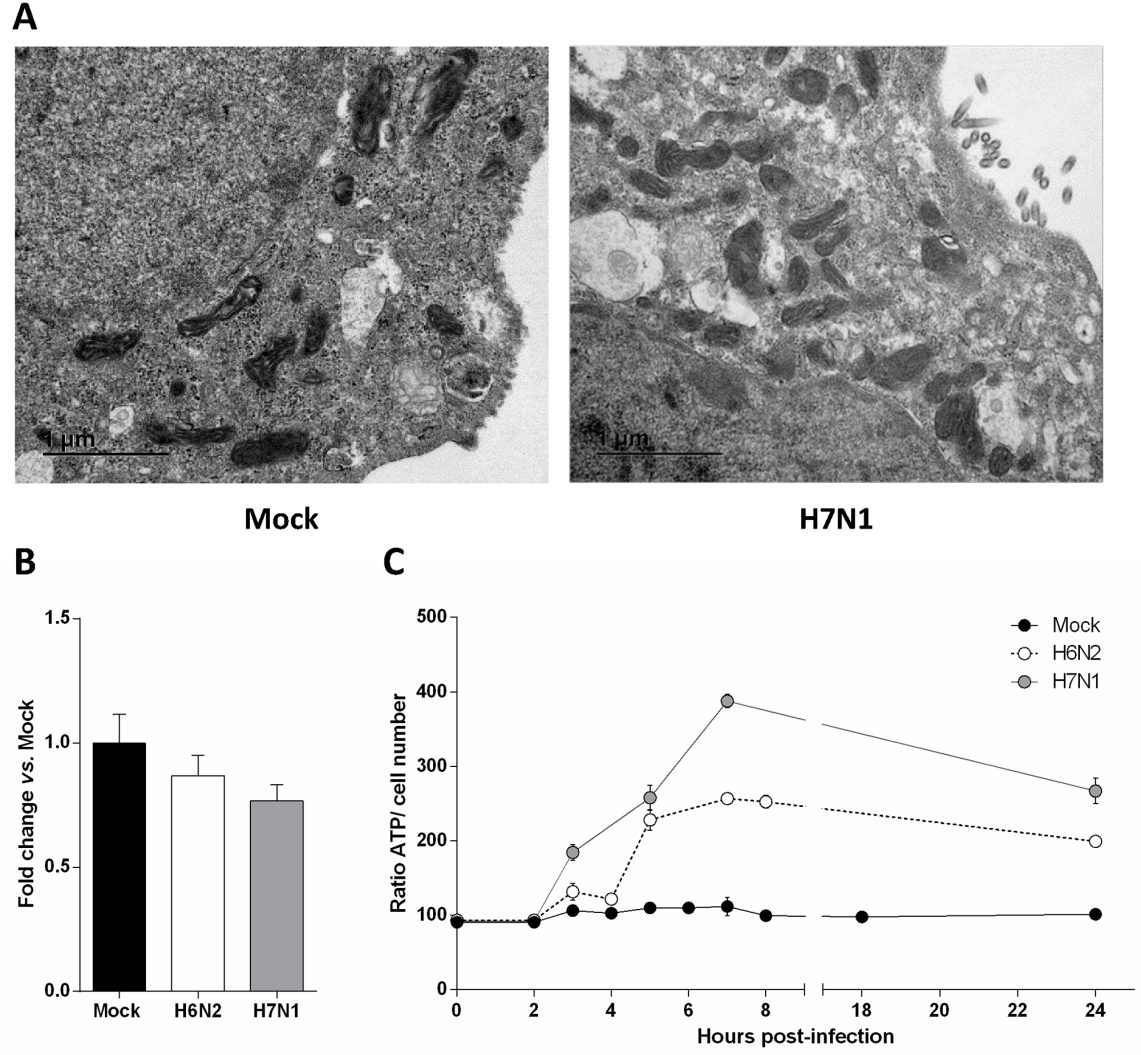
AIV infection of CLEC213 cells induces an increase in ATP production. (A) Electron microscopy analysis of H7N1-infected CLEC213 cells. Experiments were done two times independently using new batches of infected cells. Pictures are representative of 30 cell sections analyzed for mitochondria ultrastructure and number in infected or mock cells. (B) Mitochondrial DNA was quantified by qPCR in mock-, H6N2- and H7N1-infected cells. Cells were infected for 16 h at an MOI of 1. Results are expressed as fold-change vs. mock. (C) ATP production was quantified in mock-, H6N2- and H7N1-infected cells and expressed as a ratio of ATP signal on cell number. Results are shown as means ± SEM.

Because mitochondria are dynamic organelles constantly undergoing fusion and fission, we next investigated whether the observed effect could be due to an increase in the number of mitochondria. We first used electron microscopy to estimate the number of mitochondria and to characterize their typical ultrastructural features at 7 hours pi (Fig 5A). The mock- and H7N1-infected cells both presented a mean of twenty mitochondria per cell section and no mitochondrial damage or ultrastructural change could be seen in both conditions. We also quantified the mitochondrial genome copy number by qPCR and found no difference between mock and infected cells (Fig 5B). To functionally characterize the transcriptomic effects mediated by AIV infection of CLEC213 cells, we quantified the total cellular ATP produced in mock and infected conditions. As shown in Fig 5C, both H6N2 and H7N1 infections of CLEC213 cells induced a clear ATP increase in comparison to mock-infected cells. This result indicates that the infection of CLEC213 cells with AIV induces an increased activity of the oxidative phosphorylation metabolic pathway at both transcriptional and functional levels. Taken together, these results suggest that the observed increase in oxidative phosphorylation metabolic pathway is due to a real transcriptomic regulation and not to an increase in the number of mitochondria. This transcription regulation leads to a boost of ATP production within the infected cells.

### Investigation of ATP as a danger signal

**Fig 6:**
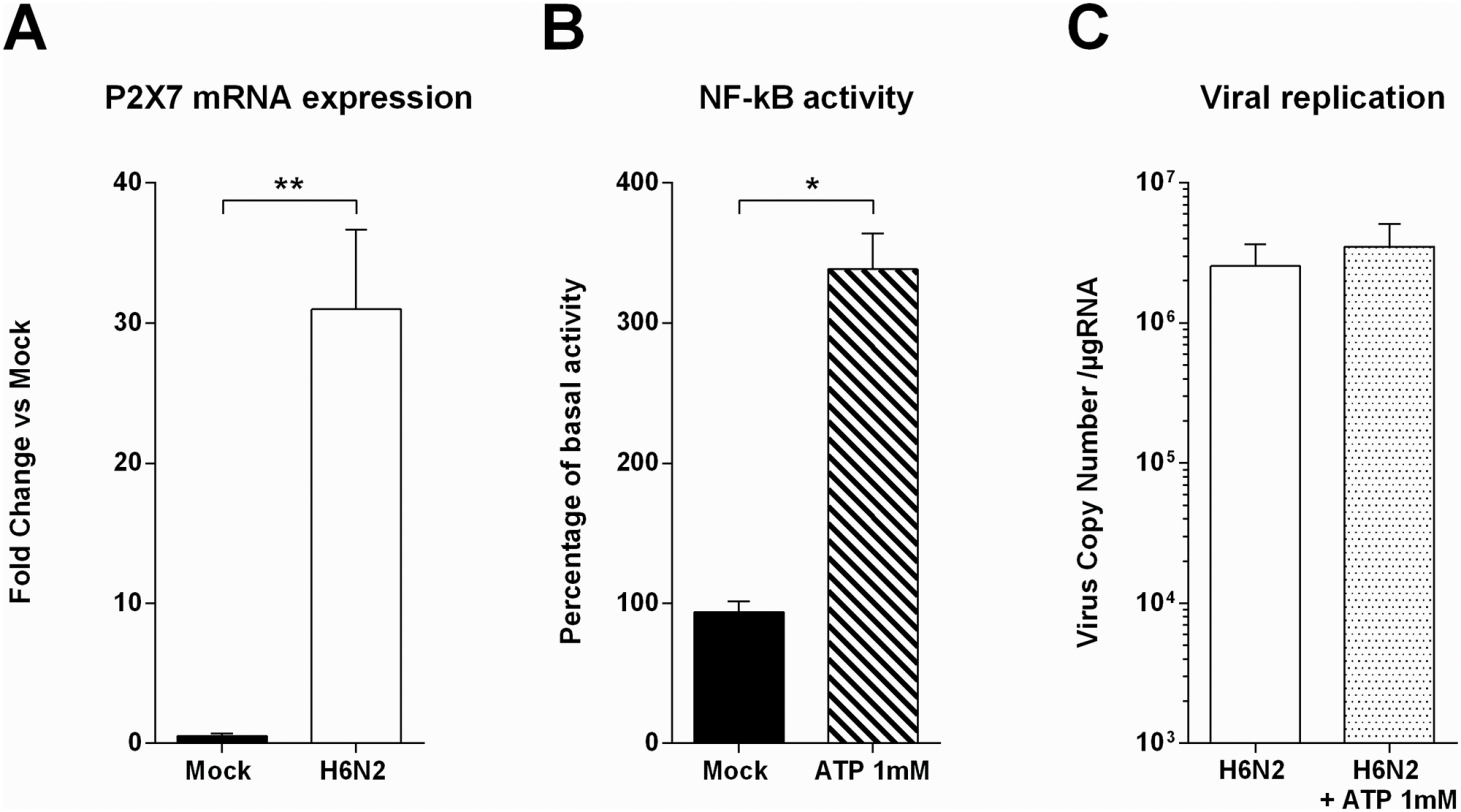
Investigation of extracellular ATP as a danger signal in CLEC213 cells. (A) P2X7R expression was measured in H6N2-infected cells by qRT-PCR. Cells were infected for 16 h at an MOI of 1. Data are means ± SEM of triplicate qRT-PCR, ** p-value<0.01. (B) CLEC213 cells were cotransfected with NF-κB -luciferase and pRSV-β-Galactosidase reporter plasmids. Cells were then stimulated with 1mM ATP for 16 h. Cell lysates were prepared and assayed for luciferase activity. Results are expressed as mean relative luminescence units normalized to β-galactosidase activity of 3 independent samples. One representative experiment of 3 is shown, * p-value< 0.05. (C) H6N2 AIV Replication was measured in presence or absence of 1mM extracellular ATP. CLEC213 cells were infected at an MOI of 1. Total RNA was extracted 16 h pi and segment 7 vRNA was quantified by qRT-PCR. Results are expressed as copy numbers per μμg of total RNA.

Extracellular ATP serves as a danger signal and is known to cause IL-1β and IL-18 processing and release [12, 22]. This inflammatory cytokines secretion is mediated through the activation of the P2X7 receptor, which exerts a potent role in the innate immune response in macrophages and myeloid cells [23]. We then investigated whether CLEC213 cells could use extracellular ATP as a danger signal to counteract AIV infection. We first measured the expression levels of P2X7 receptor by qRT-PCR in mock- and H6N2-infected cells. P2X7 is expressed by CLEC213 cells (microarray data, not shown) and highly induced by AIV infection, suggesting a potential role in the anti-viral response mounted by the CLEC213 cells (Fig 6A). In order to control the functionality of P2X7 receptor in our chicken epithelial cell line, we transfected CLEC213 cells with a construct allowing the expression of luciferase under the control of a NF-κB promoter. Cells were then treated with 1mM ATP or left untreated during 16h. As shown in Fig 6B, the ATP-stimulated CLEC213 cells displayed an increased NF-κB activity, indicating that extracellular ATP can be detected and integrated as a danger signal. To further investigate the potential impact of this ATP-mediated inflammatory pathway on the AIV life cycle, we quantified the AIV replication in presence or absence of extracellular ATP (Fig 6C). Despite a major NF-κB induction mediated by extracellular ATP, no differences could be evidenced in term of AIV replication between ATP-treated and non-treated cells. We then measured extracellular ATP production by infected cells but observed only a slight increase of extracellular ATP levels compared to the mock-infected cells, which might not be sufficient to activate P2X7 (data not shown). Collectively, these results demonstrate that extracellular ATP can be sensed by chicken epithelial cells to induce an inflammatory response. However, this response is not sufficient to counteract AIV replication.

### Inhibition of the oxidative phosphorylation metabolic pathway impacts AIV replication

**Fig 7:**
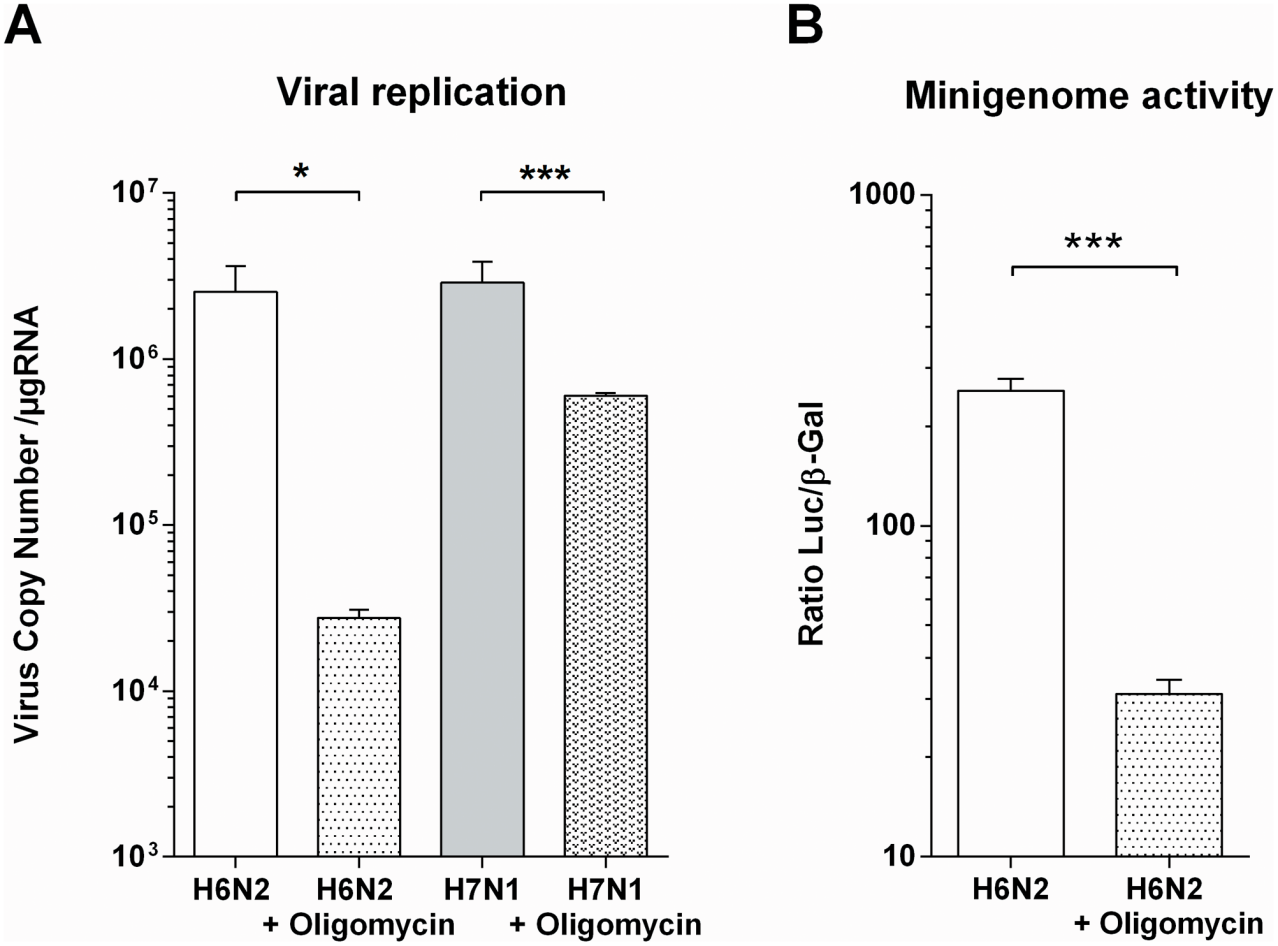
Inhibition of oxidative phosphorylation reduces AIV replication. (A) Replication of the H6N2 and H7N1 AIV was measured in presence or absence of 125μμM oligomycin. CLEC213 cells were infected at an MOI of 1. Total RNA was extracted 16 h pi and segment 7 vRNA was quantified by qRT-PCR. Results are expressed as copy numbers per μμg of total RNA, *: p-value<0.05, ***: p-value<0.001. (B) Minigenome activity in H6N2-infected cells in the presence or absence of oligomycin (125μμM). Results are expressed as a ratio of RLU on β-Gal activity ± SEM, ***: p-value<0.001.

Since ATP-mediated danger signal does not inhibit AIV replication, we speculated that the ATP production boost could be induced by the virus in order to maintain the high metabolic state necessary for viral particles production. To further delve into this hypothesis, we used oligomycin A, an inhibitor of ATP synthase. The administration of this drug (125 nM) to CLEC213 did not induce any cytopathic effect, probably due to a bioenergetic dependence switch from oxidative phosphorylation to glycolysis [24]. When oligomycin was added to the cell culture medium at the beginning of AIV infection, viral replication was drastically reduced as shown in Fig 7A. This potent inhibitory effect is also seen in a minigenome assay using H6N2 as a helper virus (Fig 7B). Thus, our data demonstrate that AIV are dependent on the oxidative phosphorylation to optimize their replication.

## Discussion

A complex interplay exists between AIV life cycle and mitochondrial functions. This interconnection is particularly obvious for MAVS-mediated innate antiviral pathways or for programmed cell death induction [9]. In any case, the main function of the mitochondrion is to orchestrate the production of ATP in the cell. Cellular energy requirements are provided by three metabolic pathways: β-oxidation, tricarboxylic cycle, and oxidative phosphorylation. The oxidative phosphorylation metabolic pathway, consisting of the respiratory chain complexes I to IV and the ATP synthase, produces about 90% of the cellular ATP [25]. In the present study, with the aim of characterizing the host response of chicken respiratory epithelial cells to AIV, we identified a clear increase in the mitochondrial genes encoding proteins of the oxidative phosphorylation metabolic pathway. This mitochondrial signature was confirmed using qRT-PCR and the metabolic functional consequences were also validated by ATP assays. Similar results have been described by Wu and collaborators: using a proteomic approach, the authors of the study described an induction of different component of the oxidative phosphorylation during the infection of A549 cells by a swine H3N2 influenza virus [26].

A striking feature of the mitochondrial transcriptomic signature identified in our work is that all the components of the oxidative phosphorylation encoded by the mitochondrial genome are overexpressed under AIV infection. This feature can be explained by the prokaryotic origin of the mitochondrion. Indeed, once mitochondrial transcription is initiated, the DNA strands are transcribed as polycistronic precursors RNA, including all genetic information encoded by the mitochondrial genome [19]. Transcription enhancement thus increases all the genes encoded by the mitochondria. The origin of this transcriptional enhancement is unknown. We first hypothesized that this mitochondrial transcriptomic effect of AIV infection could be due to the expression of the small protein PB1-F2. Indeed PB1-F2 has a strong mitochondrial tropism and impacts the mitochondrial functions [20, 21, 27, 28]. Yet, on the contrary, its expression seems to decrease the mitochondrial genes expression (Fig 4C). Consequently, PB1-F2 is probably not involved in this mechanism. These results were surprising since we previously identified a PB1-F2-associated mitochondrial signature in H5N1-infected chicken blood cells. However, the mitochondrial effect of PB1-F2 was not found in lungs of infected chickens, and could be specific for certain types of cells [17].

Our study shows a clear increase in ATP production in AIV-infected cells. As part of an innate defense mechanism, cells can detect extracellular ATP as a danger signal using a variety of purinergic receptors, including purinergic receptor P2X7 [10]. ATP binding to P2X7 receptor leads to a cation efflux, cytokines production and Prostaglandin E2 (PGE2) secretion. As a result, this activates the NLR family pyrin domain containing 3 (NLRP3) inflammasome pathway in mammalian models [29]. It has been described that chickens express NLRP3, caspase I and IL1-β [30–32], though the latter lacks a caspase I cleavage site [33], indicating that there might be slight differences in inflammasome signaling between mammals and other vertebrates. Most of the research on P2X7 involvement in immune response were made on immune cells. However, Huang and co-workers described a role for P2X7 signalization in epithelial cells leading to cytokine production independently of the inflammasome [34]. In addition, Lee *et al*. worked with an impaired-inflammasome model suggesting that ATP can act as a modulator of the immune response via P2X7 activation leading to IL-6 production [10]. Our results show that CLEC213 cells express P2X7 receptor and that its expression is increased in AIV-infected cells (Fig 6A). Furthermore, ATP-treated cells show a NF-κB pathway activation (Fig 6B) which may be linked to P2X7 activity. However, when measuring extracellular ATP produced by AIV-infected cells, we detected only femtomolar concentrations of ATP (not shown), when it has been described that ATP can activate purinergic receptor at millimolar concentrations [12]. We failed to detect any direct protective effect when we added extracellular ATP at the millimolar level on AIV-infected cells (Fig 6C), suggesting that ATP alone is not sufficient to help epithelial cells mount an efficient response against AIV. Studies on P2X7 in epithelial cells suggested a role in enhancement of inflammation and immune cells recruitment. Such effect is obviously not visible in our epithelial-only cell model.

AIV, as an obligate intracellular parasite, is dependent on the metabolic capacities of its host, and increasing ATP production of the infected host could be part of a viral strategy to boost replication capacities. Such strategy has previously been described for other viruses like vaccinia virus which induce the expression of two mitochondrial genes: Mitochondrially encoded NADH dehydrogenase subunit 4 (MT-ND4) and Mitochondrially Encoded Cytochrome C Oxidase II (MT-Co2) [35]. As a result, intracellular ATP generation was increased in infected Hela cells, with a beneficial effect for the virus. Surprisingly, this observation could not be extended to AIV-infected MDCK [35]. Finally, we sought to demonstrate that this ATP increase could be beneficial for AIVs. The massive amounts of energy generated by the cell could be used by the virus to support its life cycle, for instance viral protein synthesis and assembly. This has been shown for many DNA viruses such as Human herpesvirus 8 and other oncoviruses that modulate the metabolism of the host cell [36, 37]. A similar effect was also observed with an RNA virus, namely the Dengue virus [38]. In our study, we showed that the AIV infection resulted in greater oxidative phosphorylation activity. When we blocked the ATP production by adding oligomycin (an inhibitor of the F0 part of H+-ATP-synthase), we were able to observe an impaired viral replication in the early times post-infection (Fig 7), underlining the importance of metabolic needs for the virus to efficiently invade the cells.

Collectively, these data suggest that upon AIV-infection, chicken lung epithelial cells are able to secrete inflammatory cytokines but are highly susceptible to infection. Indeed, Influenza virus seems to be able to deeply modify cell metabolism via the stimulation of the oxidative phosphorylation pathway, leading to an increase in ATP production which seems to benefit the virus. In spite of a functional P2X7 receptor and ATP-dependent NF-κB activation, the increase in ATP production seems to remain in the intracellular compartments, preventing efficient ATP signaling. However, it cannot be excluded that in the context of a whole organ, epithelial cells could contribute to global inflammation leading to the recruitment of myeloid cells to the site of the inflammation.

## Supporting information

S1 Table Details of primers used in this study. (XLSX)

S1 Fig Correlation between quantitative PCR of viral copy number and virus titration. MDCK cells were infected with different doses of IAV and subjected to plaque assays for virus titration. The supernatants of the same samples were used to extract viral RNA. After reverse transcription, cDNA were used to determine viral M1 copy number (i.e. segment #8). Data obtained were analyzed using the Pearson r correlation test in order to verify the interrelationship between the 2 quantification methods.
(PDF)

S2 Fig Correlation between quantitative RT-PCR and microarray data. CLEC213 cells were infected by H6N2 virus at a MOI of 2. At 16 hours post-infection, total RNA was extracted from cells and analyzed by microarray or by RT-qPCR. The table provides the mean of data obtained for 4 replicates in each methods. The scatter plot represents the distribution of the gene expression depending on the method used for quantification. The scatter plot representation indicates that RT-qPCR quantification slightly overestimates the gene expression when compared to the microarray quantification.
(PDF)
