# Supplementary material for: Transcriptomic profiling of a chicken lung epithelial cell line (CLEC213) reveals a mitochondrial respiratory chain activity boost during influenza virus infection
Source: PLoS One. 2017 Apr 25;12(4):e0176355. doi: 10.1371/journal.pone.0176355 (PMC5404788; doi:10.1371/journal.pone.0176355)
Supplement: S1 Fig — MDCK cells were infected with different doses of IAV and subjected to plaque assays for virus titration. The supernatants of the same samples were used to extract viral RNA. After reverse transcription, cDNA were used to determine viral M1 copy number (i.e. segment #8). Data obtained were analyzed using the Pearson r correlation test in order to verify the interrelationship between the 2 quantification methods. (PDF) [file pone.0176355.s002.pdf]

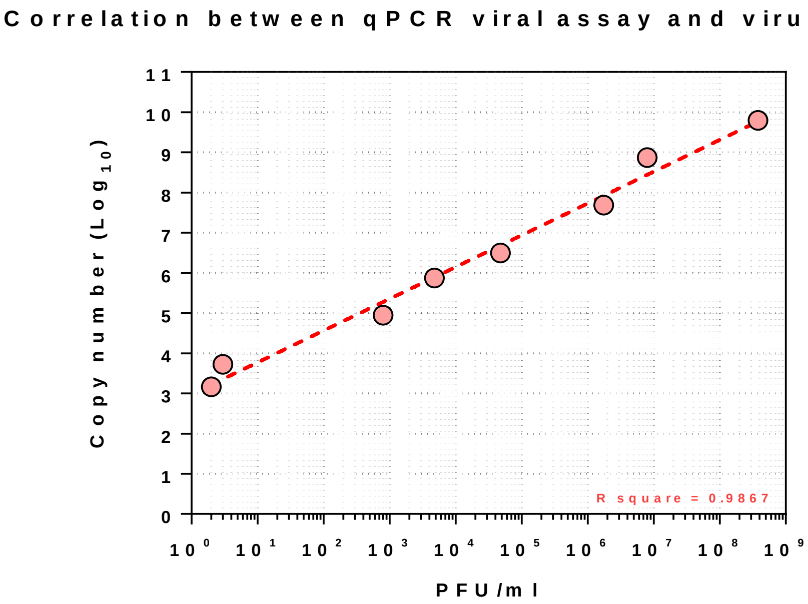


| Pearson r | |
|---|---|
| r | 0.9952 |
| 95% confidence interval | 0.9729 to 0.9992 |
| R square | 0.9905 |
| | |
| P value | |
| P (two-tailed) | < 0.0001 |
| P value summary | **** |
| Significant? (alpha = 0.05) | Yes |

**Supplemental Figure 1 : correlation between quantitative PCR of viral copy number and virus titration.**
MDCK cells were infected with different doses of IAV and subjected to plaque assays for virus titration. The supernatants of the same samples were used to extract viral RNA. After reverse transcription, cDNA were used to determine viral M1 copy number (*i.e.* segment #8). Data obtained were analyzed using the Pearson r correlation test in order to verify the interrelationship between the 2 quantification methods.
