# Supplementary material for: Transcriptomic profiling of a chicken lung epithelial cell line (CLEC213) reveals a mitochondrial respiratory chain activity boost during influenza virus infection
Source: PLoS One. 2017 Apr 25;12(4):e0176355. doi: 10.1371/journal.pone.0176355 (PMC5404788; doi:10.1371/journal.pone.0176355)
Supplement: S2 Fig — CLEC213 cells were infected by H6N2 virus at a MOI of 2. At 16 hours post-infection, total RNA was extracted from cells and analyzed by microarray or by RT-qPCR. The table provides the mean of data obtained for 4 replicates in each methods. The scatter plot represents the distribution of the gene expression depending on the method used for quantification. The scatter plot representation indicates that RT-qPCR quantification slightly overestimates the gene expression when compared to the microarray quantification. (PDF) [file pone.0176355.s003.pdf]

	Gene	Fold changes *vs.* mock Microarray	Fold changes *vs.* mock RT-qPCR
**Mitochondrial genes**	MT-ATP8	2,9	8,4
	MT-ATP6	3,9	8,3
	MT-CO2	4	5,2
	MT-COI	4,4	6,4
	MT-CO3	3,5	6,1
	MT-CYB	4,2	8,3
	MT-ND1	2,4	10,6
	MT-ND2	3,9	4,8
	MT-ND3	6,2	6,2
	MT-ND4	4,8	10,8
	MT-ND5	5,6	12,4
**Nuclear genes**	STAT1	-2,3	-2,0
	CD93	-1,1	-1,6
	TRAF5	-2,0	-1,6
	IL8	3,9	2,8
	TLR7	0,9	1,3
	IFNA3	3,1	5,8

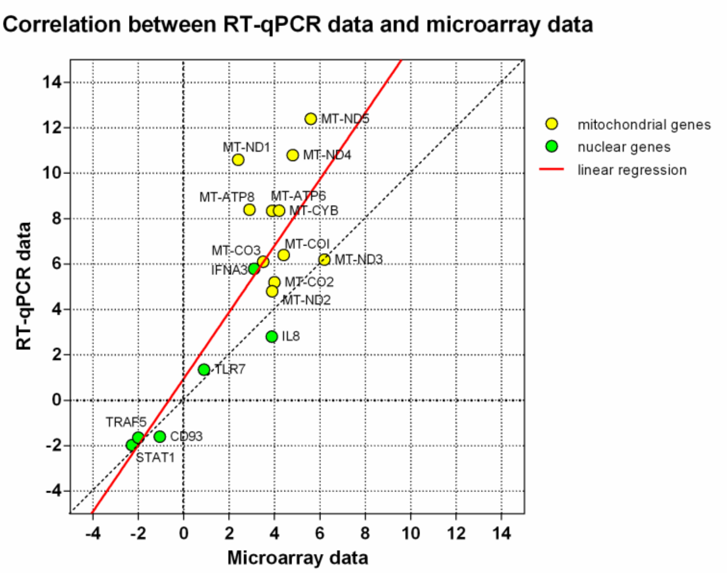

Supplemental Figure 2 : correlation between quantitative RT-PCR and microarray data.
CLEC213 cells were infected by H6N2 virus at a MOI of 2. At 16 hours post-infection, total RNA was extracted from cells and analyzed by microarray or by RT-qPCR. The table provides the mean of data obtained for 4 replicates in each methods. The scatter plot represents the distribution of the gene expression depending on the method used for quantification. The scatter plot representation indicates that RT-qPCR quantification slightly overestimates the gene expression when compared to the microarray quantification.
